# Age-Sensitive Effects of Enduring Work with Alternating Cognitive and Physical Load. A Study Applying Mobile EEG in a Real Life Working Scenario

**DOI:** 10.3389/fnhum.2015.00711

**Published:** 2016-01-13

**Authors:** Edmund Wascher, Holger Heppner, Sven O. Kobald, Stefan Arnau, Stephan Getzmann, Tina Möckel

**Affiliations:** IfADo-Leibniz Research Centre for Working Environment and Human FactorsDortmund, Germany

**Keywords:** working environment, mental fatigue, mobile EEG, aging

## Abstract

Ergonomic assessment of a workplace requires the evaluation of physical as well as cognitive aspects of a particular working situation. In particular the latter is hardly possible without interfering in the natural setting. Mobile acquisition of neurophysiological measures (such as parameters of the EEG) may close this gap. At a simulated workplace we tracked older and younger participants with mobile EEG during a 4–5 h work shift. They had to perform either a monotonous cognitive task, a self-paced cognitive task or a self-paced physical task in a predefined order. Self assessment, behavioral performance and spectral measures of the EEG (before most alpha power) indicated that younger participants suffered from monotony. Older adults, on the other hand, were overall impaired by inefficient information processing. This was visible in EEG variations time-locked to eye blinks (blink-related synchronizations), a new measure to investigate cognitive processing in real life environments. Thus, we were able to distinguish between active and passive task-related aspects of mental fatigue without impinging on the natural working situation.

## Introduction

Evaluation of workplaces may take place on quite different levels. Traditional ergonomics focuses on physiological and physical factors of working environment, applying surveys to different aspects of the workplace in order to support and protect the worker (McAtamney and Corlett, 1993; Hignett and McAtamney, 2000; Halim et al., 2012). More recently, virtual models of humans help to evaluate many of these aspects of work (Lämkull et al., 2007; Bandouch et al., 2008). Cognition, however, and human factors affecting cognition, can be addressed only superficially in real life situations. While experimental psychology may address distinct cognitive aspects that play a role in working situations, this can hardly apply for the complex interaction of cognitive requirements that a worker faces during a work shift. Neurophysiological measures that can be taken while regular work is performed may help to close this gap. In particular, the increasing accessibility of mobile neurophysiological technology that allows for an “online” registration of work-related parameters is a huge challenge and chance for ergonomic evaluation (Wascher et al., 2014a).

In the present study, we focused on a neuroergonomic evaluation of the interaction between mental fatigue and age-related changes in cognitive performance. The proportion of employed older adults is continuously increasing with the general demographic change. While older employees may benefit in many cases from professional experience, they are also facing physiological and cognitive decline, which makes it difficult to keep up with their younger colleagues, in particular when long-term working situations are considered. There is some evidence from laboratory experiments that not only physical but also mental fatigue raises faster with higher age (Wascher and Getzmann, 2014). On the other hand, it has been shown that varying the cognitive task may prevent older adults from accelerated raise of mental fatigue (Falkenstein et al., 2002). However, does this finding hold also for situations in which varying tasks require flexible retrieval of cognitive and physical capabilities continuously, as it may be the case during a working shift?

We addressed this question based on a multi-level model of fatigue and its possible relation of age-related cognitive decline by pinning the observed behavior measures down to neurophysiological mechanisms.

Long lasting activity, independently whether it is physical or cognitive, leads to a decline in capabilities of a worker (Halim et al., 2012; Lerman et al., 2012). Despite this communality and the usage of the common term of “fatigue,” physical and cognitive declines have quite different underlying mechanisms and consequences. Physical fatigue goes along with metabolic changes in the muscle, which leads a decline in physiological capabilities. Additionally, an increase in the plasma fatty acid level may lead to an increase in tryptophan. This is assumed to increase the 5-HT concentration in the brain and thereby contributing to central fatigue (Newsholme et al., 1992), a mental contribution to physical fatigue. Although central aspects may play an important role for the accessibility of physical resources (Marcora et al., 2009; Mehta and Parasuraman, 2013), the peripheral exhaustion of resources is the core aspect of physical fatigue.

Mental fatigue, on the other hand, is not known to go along with any physiological resource consuming aspect (Hockey, 2013). Apart from circadian rhythms and sleeping behavior (sleep-related fatigue = SR), mental fatigue may derive either from cognitive overload (active task-related fatigue = aTR) or from mental underload (passive task-related fatigue = pTR) due to, e.g., monotony (May and Baldwin, 2009). Those latter two mechanisms end up in distinct types of fatigue that have different consequences for subsequent activity. While mental aTR was found to persist even after completion of the task and not to depend on the amount of motivation (van der Linden et al., 2003), pTR is strongly coupled with the motivational system (Boksem et al., 2006; Boksem and Tops, 2008; Bonnefond et al., 2011). Both in laboratory basic research studies as well as in applied contexts it was demonstrated that pTR can be efficiently effaced by incentives. Even after long lasting mental tasks, the instruction to put more effort into a given task is efficient to restore cognitive performance almost completely to the level that has been shown at the beginning of the experiment (Boksem et al., 2006). Therefore, pTR has been framed in motivational terms. Boksem and Tops (2008), for example, proposed that pTR reflects an imbalance between resources invested and outcome. Whenever the effort that is needed to perform on an adequate level is too high in relation to the outcome that is generated, motivation declines and, as a consequence, cognitive processing becomes less efficient.

Until now, the different aspects of mental fatigue have been investigated in isolation and under controlled laboratory settings. However, one has to be aware that most working activities that require physical and cognitive effort also contain periods in which monotonous cognitive tasks have to be performed. Such changes in duties may prevent at least from pTR, as has been demonstrated in studies that investigated effects of aging on mental fatigue. When tasks changed during the experimental session no mental fatigue was observed in older adults (Falkenstein et al., 2002), whereas performing the same task for a longer period of time led to a clear age-related decline of attentional performance (Wascher and Getzmann, 2014).

Age-related effects on pTR are insofar of central interest in fatigue research as the well-known decline of structures in the frontal lobe of the human brain (Chao and Knight, 1997) affects primarily those structures that are also involved in motivational processes and thereby in upholding cognitive performance in non-demanding situations (Berridge and Robinson, 1998). Beside this, lower muscle strength with higher age may contribute to central fatigue when physical demanding tasks are part of a working situation.

We designed a simulated workplace that resembled the post room of a German wholesale house (see Funding). The tasks of the participants recreated parts of the real workflow but were nevertheless controlled experimental settings. Participants had to perform a monotonous stimulus-response task, a self-paced cognitive task, and a physical task (moving and sorting boxes of different weights and sizes) in a repetitive sequence for about 4–5 h. Behavioral performance was measured in the cognitive tasks and self estimation of experienced fatigue and motivation were repeatedly taken. One of the core questions of the present study was, to what degree a neuroergonomic approach may help to get objective data from task load, effort, and fatigue-related changes in cognitive processing. To this end, beside the “classical” measures of behavioral performance and self estimation, mobile EEG was recorded continuously, while the participants were freely moving in an office-like room, dealing with the different tasks.

Since there is hardly any literature to such experimental setting, we focused firstly on well-known aspects of the EEG that have been related to mental fatigue. This includes before most oscillatory activity. It has been repeatedly shown that brain oscillations are slowing down with mental fatigue, indicated by increasing power in the alpha and the theta band of the EEG (Lal and Craig, 2001; Akerstedt et al., 2004; Wascher et al., 2014b). Given that most of these studies used longer lasting monotonous tasks, these effects may be related before most to pTR. In particular, the increase in alpha activity may well be a correlate of decreasing motivation and a withdrawal of attention that lead to a kind of idle state in the sensory and attention related structures of the brain (Hanslmayr et al., 2012) that should be most pronounced in the monotonous stimulus-response task. The increase in theta activity may be rather related to increasing effort that is invested to keep performance high (Sarter et al., 2001, 2006) which should be stronger in self-paced tasks. Most interestingly, a kind of slowing has been also reported with increasing age, as indicated by a reduction in the individual alpha frequency (for a review see Klimesch, 1999), but so far not for mental fatigue.

Besides these rather energetical aspects of brain activity, we asked for specific neuronal processes that can be related to information processing, and how they change with age and fatigue. In laboratory settings, stimuli are presented at distinct time points and cortical activity is measured time-locked to these events (so-called event-related potentials or oscillations). Such events are not accessible in real life situations. Adding additional stimuli to a real life situation may substantially alter the task of participants, leading to, e.g., attentional distraction. Distinct events from the surrounding that may be identified by scene cameras would be not comparable across different tasks, and no sufficient number of repetitions of comparable events is guarantied. Events that occur independently of a particular task, repetitively also in real life situations are so-called eye events. Horizontal eye-movements (e.g., saccades) are the core human behavior related to spatial orientation of attention. More interesting for the temporal segmentation of incoming information are eye blinks that occur primarily at the end of an information processing sequence (e.g., Doughty, 2001; Wascher et al., 2015). Recently, we demonstrated that time locking of EEG activity to eye blinks provide reliable measures for cognitive effort (Wascher et al., 2014a). Because event-related potentials did not reliably show time on task related changes in a previous task (see Wascher and Getzmann, 2014), we applied event-related synchronization/desynchronization (ERS/ERD; Pfurtscheller and Aranibar, 1979) analyses to the eye-blink related data. Due to the lower time resolution, these data might be more robust in complex experimental situations like a workplace simulation. Moreover, phasic changes in brain oscillatory activity (in particular in the Alpha band) appear to be reliable correlates of signal processing (Klimesch et al., 2002; Müller et al., 2009).

Taken together, applying these methods to a working situation that resembles a real workplace should provide information about age and fatigue-related changes in information processing and the underlying neuronal mechanisms. The aim of the present study was to go beyond the description of age-related differences in performance.

## Methods

### Participants

Thirteen younger adults (20–29 years old, mean age 25.3) and 12 older adults (55–73 years old, mean age 64.4) took part in the experiment. All participants had normal or corrected to normal vision, were of good physical health, and reported no history of psychiatric or neurological diseases. For the entire procedure (lasting around 5–6 h including preparation) participants received 60 €.

Prior to the experiment participants gave written informed consent. The study was approved by the local ethics committee and according to the Declaration of Helsinki.

### Task, stimuli, and procedure

**Figure 1:**
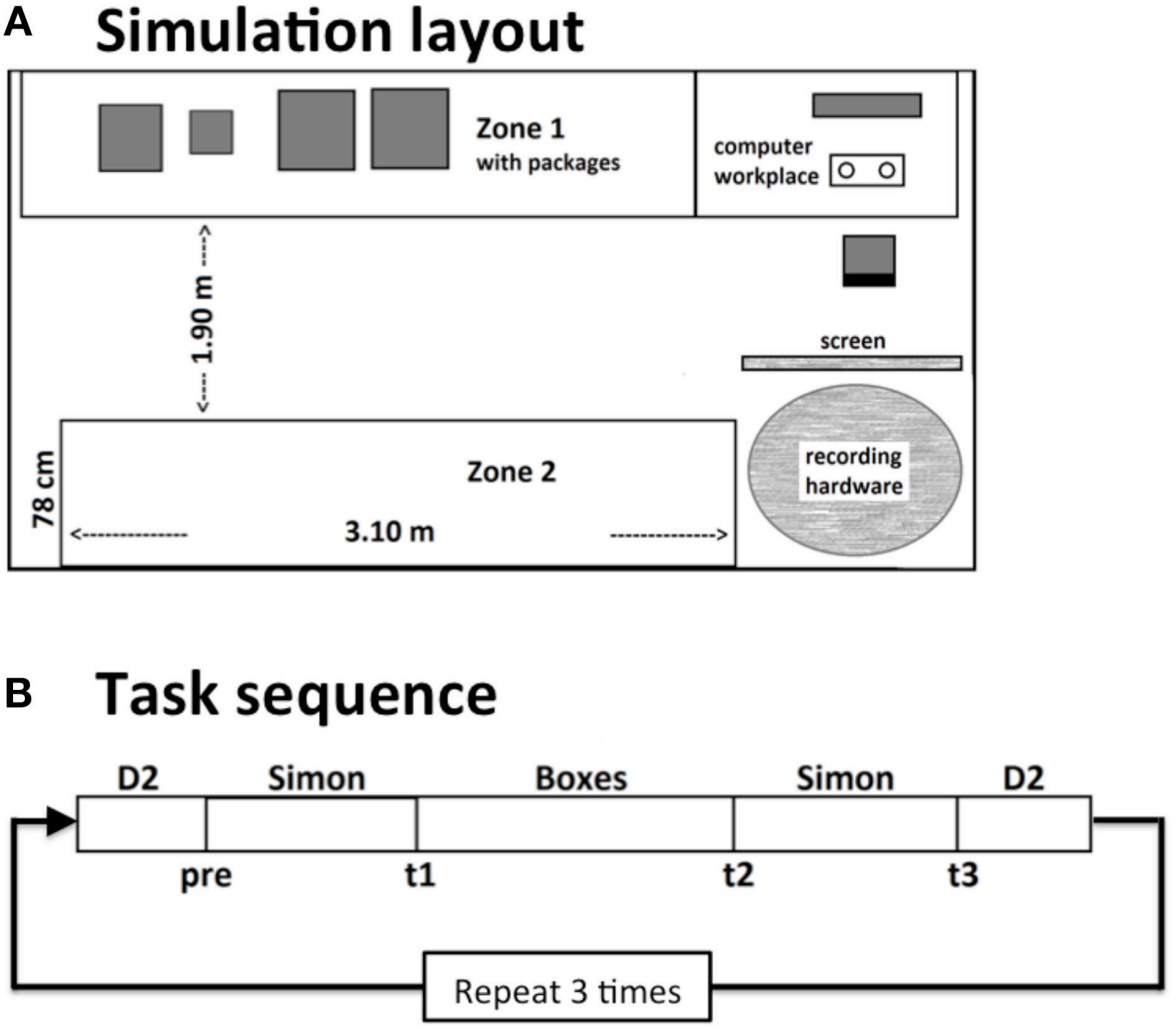
**Schematic layout of the workplace simulation (A)**. In an office-like room, participants either had to perform a monotonous or a self-paced cognitive task at a computer. In between they had to move boxes between two zones. Thereby they had to follow randomly changing sorting instructions. This procedure was repeated 3 times in a 4–4.5 h lasting work shift **(B)**.

The experiment took place in an office room (3.50 × 4.80 m) with partly covered windows. Tables stood along the walls where the boxes for the physical task were placed (see Figure 1). On one of the tables, a computer monitor was positioned for the presentation of instructions for the physical task and the cognitive tasks. A research assistant who controlled the experiment sat in another room and monitored the EEG recordings. Participants could reach the assistant via phone at all times. The assistant only entered the room during breaks or to correct any technical failures if necessary.

The experiment consisted of three tasks, which were repeated in a predefined sequence within each block. Blocks started and ended with a computerized version of the d2-task, that was closely oriented on the original paper-pencil version (Brickenkamp, 1962). In between, a block of the Simon task was presented, followed by the physical task and again the Simon task had to be performed. This procedure was repeated three times with short breaks in between and added up to an overall duration of the work shift of about 4–4.5 h. Each subtest was defined for a pre-defined duration, thus the self-paced tasks were stopped no matter how many passes were finished.

In the d2-task, three lines of 57 d's or p's each with one to four marks (in the form of single or double quotes) above and/or below the letter were presented. The participants had to mark as much d's with exactly two marks as possible in a given time window using a computer mouse, while simultaneously ignoring all p's and the d's with less or more than two marks. The participants had 20 s per line. Then a sound signaled to proceed with the next line. After the three lines were done, a new screen appeared with three new lines. In each d2-task block, five screens with three lines each were presented. Overall, one d2-task block took about 5 min. The d2-task served as a self-paced cognitive task.

In the Simon task (Simon, 1969) one of two symbols (either a square or a diamond) was presented on the left or the right side of a fixation cross for 150 ms. The participants had to decide which one of the symbols was shown by responding with either the left or the right hand, while ignoring the side on which the stimulus was presented. Thus, a trial could be either corresponding (stimulus presentation and response on the same side) or non-corresponding (stimulus presentation and response on different sides). The inter-stimulus interval was 1800 (±500) ms, and 704 stimuli were presented overall. Each block of the Simon task took about 21 min. The Simon task served as an externally-paced, monotonous cognitive task.

Before and after each Simon task the participants were asked to rate their subjectively experienced amount of mental fatigue and their motivation to continue with the task on a 9-point Likert scale.

In the physical box-sorting task (Boxes), participants had to handle 12 cardboard boxes of three different sizes and three different weights (0.5–15 kg). The boxes were placed on waist high tables, which formed two “zones” on opposite sides of the room. The distance between the two opposite zones was about 190 cm. Participants had to carry the boxes between these zones. They had to sort them according to size, weight, or label, consisting of either a letter (A, B, C) or a number (1–12) both attached to the boxes. Boxes always had to be arranged in three groups of four objects each. In case of sorting by numbers, boxes 1–4, 5–8, and 9–12 had to be put together. In case of sorting by letters, boxes A, B, and C had to be put together. The sorting rules were presented on a computer screen. After finishing one sorting task the participant had to press a button on the keyboard to get new instructions. The order of the sorting rules was randomized. There was no time limit for a single sorting task. Overall, one physical task block was performed for 25 min. A message on the screen signaled the ending of the block. The physical task was not paced at all.

### EEG data recording and processing

EEG was recorded from 60 standard electrode sites using an active electrode system (ActiCap; BrainProducts). Vertical eye movements and blinks were measured from two electrodes above and below the right eye (vEOG). Two electrodes at the outer canthi of both eyes were used for the measurement of horizontal eye movements (hEOG). Electrode impedance was kept below 10 kΩ. EEG and EOG were digitized at 1000 Hz and submitted via a WiFi module (MOVE; BrainProducts) to a BrainAmp MR plus EEG amplifier (BrainProducts). Data was recorded with a resolution of 0.1 μV, a Low Cutoff at DC and a High Cutoff at 250 Hz. Transmitter and the ActiCap Control Box were placed in a belt bag at the lower back of the participants. They could move around without any restrictions.

Data were offline re-referenced to averaged mastoids and a bandpass filter (0.5–45 Hz) was applied. Data were set up both for regular and event-related frequency analyses (event-related desynchronizations/synchronizazions = ERD/ERS: see Pfurtscheller and Aranibar, 1979; Klimesch, 1999; Pfurtscheller and Lopes da Silva, 1999). Because of the large structural differences between tasks, no task-inherent temporal markers for event-related analyses were available across tasks. Therefore, we referred to eye blinks as temporal marker for information processing (Wascher et al., 2014a, 2015). Only singular blinks were used for this procedure, which were not followed by another blink within 700 ms. Additionally, blinks were excluded that were accompanied by marked horizontal eye movements (for more detailed information about the blink detection mechanism, see Wascher et al., 2015). Data segments from −1000 to 2000 ms around the maximum of blink-related activity in the bipolar vEOG were extracted. An interval between −450 and −250 ms served as baseline. This interval was selected to avoid any temporal overlap with ongoing vertical eye movements. After statistics-based artifact removal as implemented in EEGLAB (Delorme and Makeig, 2004), an independent component analysis (ICA) was applied (data down-sampled to 250 Hz). Independent components (ICs) reflecting artifacts were identified and rejected using ADJUST (Mognon et al., 2011). The remaining ICs were tested for biological plausibility based on their scalp maps. The goodness of fit for modeling each IC with a single equivalent current dipole was calculated by submitting individual component maps to an automatic source localization algorithm (DIPFIT, contributed to EEGLAB by Oostenvelt et al., 2003), using a standard four-shell spherical head model. Any IC with a residual variance of more than 40% was automatically removed from the data (for a similar procedure see Debener et al., 2005).

For the analyses of frequency spectra, fast-fourier transformations (FFTs) were applied to the extracted segments, using the spectopo function of EEGLAB. In order to provide a sufficient resolution of frequencies, data were padded with zeros to a length of 2048 data points (freqfrac = 4). For ERD/ERS analyses, the matrix of valid ICs was projected back to the continuous data set for band-pass filtering (4–7.5 Hz for Theta activity; 8–12 Hz for Alpha activity). Segments that were marked as artificial in the preprocessing pipeline were removed from those data as well.

### Data analysis

#### Self assessment

Analyses of variance for repeated measurements (ANOVAs) were conducted for the subjective measures (i.e., the rated mental fatigue and rated motivation to continue the task) with the between-subject factor Age (younger, older), and the within-subject factors Time on Task (ToT; across the three blocks) and Sequence (order within each block).

#### Behavioral data (simon task)

ANOVAs were conducted for response times and error rates in the Simon task with the between-subject factor Age (younger, older) and the within-subject factors ToT, Sequence (task run before vs. after physical task), and S-R Correspondence (relates to the spatial relation between stimulus and response location: corresponding vs. non-corresponding).

#### EEG data

All EEG analyses were restricted to FCz and POz, two electrodes that are commonly reported in studies investigating mental fatigue.

Since several individuals in the sample showed either multiple peaks in the EEG spectrum or no peak at all, we chose the gravity frequency method in order to determine the individual Alpha frequency (IAF; see Klimesch, 1999). Gravity frequency (GF) is defined as the weighted sum of spectral estimates in the Alpha range divided by the total Alpha power (Goljahani et al., 2012). Extracted power measures were individually adjusted to GFs (for a review see Klimesch, 1999). Lower alpha power was defined as the mean power between GF–2 Hz and GF. Upper Alpha ranged from GF to GF + 2 Hz, Theta from GF − 5 to GF − 3 Hz and Beta from GF + 5 to GF + 18. GF and the mean power in all bands were entered into ANOVAs with the between subject factor age and the within subject factors Task (3; D2, Simon task, Boxes), Time on Task (3), and Electrode (2; FCz, POz).

For ERD/ERS analyses, band-pass filtered data were squared and set into relation to the mean power in the baseline (−1000 to 0 ms relative to the blink maximum). The most impressive effect occurred immediately after the re-opening of the eyes (see also **Figure 8**). Therefore, ERD/ERS were measured in a distinct time windows between 0 and 300 ms after the blink maximum. Mean ERD/ERS were calculated for this time window and entered into the same analysis as power values and GF.

For factors with more than two levels, Greenhouse-Geisser adjusted *p*-values are reported where appropriate. Additionally, effect sizes by means of partial eta squared ($\eta_{p}^{2}$) are reported for significant results. Post-tests were Bonferroni corrected. Signal analyses were performed on MATLAB®. All statistical analyses were conducted using GNU R (R Core Team, 2012). Plots were drawn using VEUSZ (Jeremy Sanders, 2013; http://home.gna.org/veusz/).

## Results

### Self assessment

**Figure 2:**
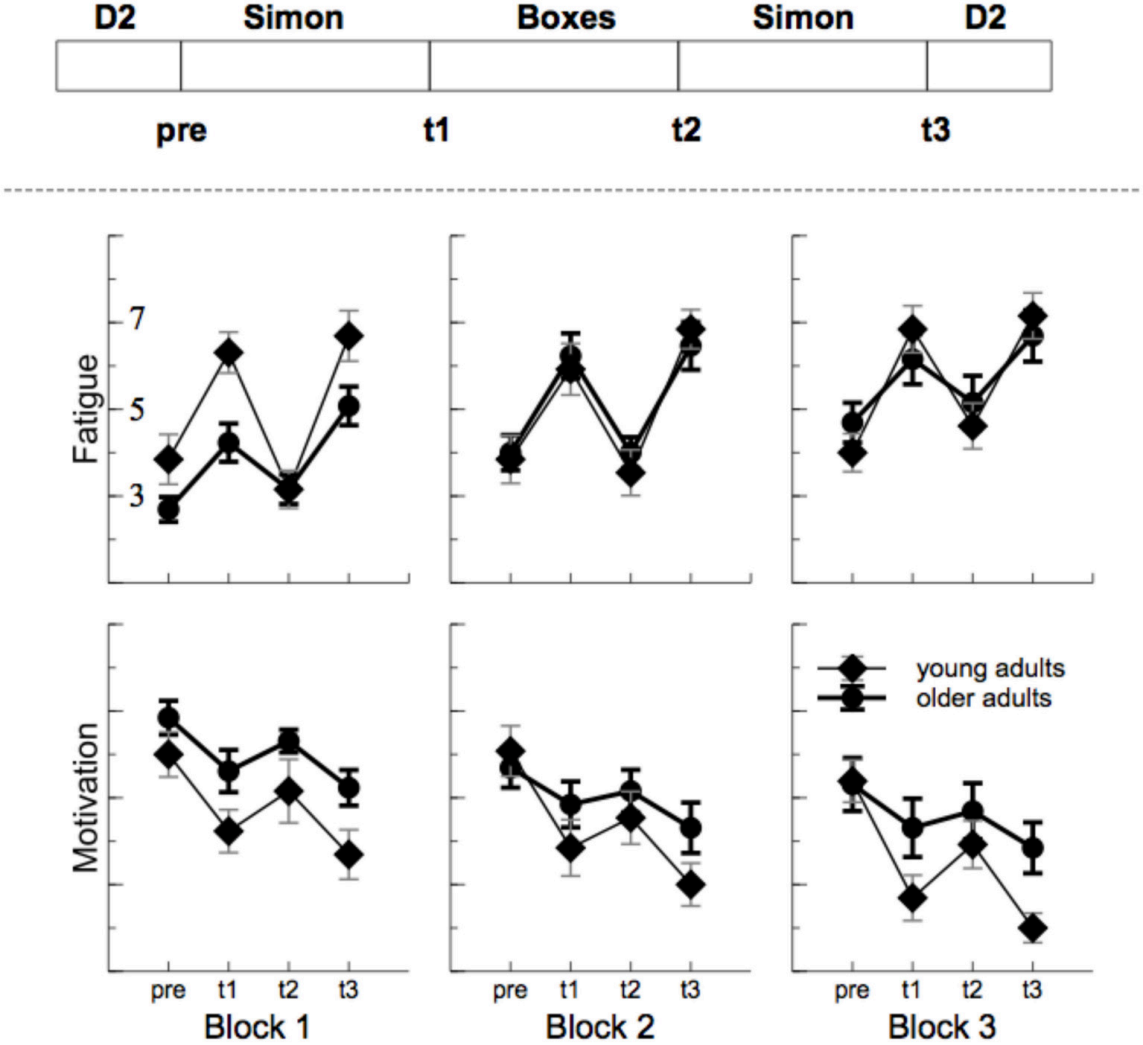
**Sequence of task and outcome of the self assessment**. Fatigue increased and motivation decreased with time on task. In particular after the monotonous Simon task (t1 and t3).

Fatigue increased with ToT, *F*_(2, 48)_ = 9.70, *p* = 0.001, $\eta_{p}^{2}=0.29$, and motivation decreased, *F*_(2, 48)_ = 13.92, *p* < 0.001, $\eta_{p}^{2}=0.37$. For both scales (see Figure 2), a clear modulation was found with the task performed [fatigue: *F*_(3, 72)_ = 56.04, *p* < 0.001, $\eta_{p}^{2}=0.70$, motivation: *F*_(3, 72)_ = 28.94, *p* < 0.001, $\eta_{p}^{2}=0.55$]. In particular, after the Simon task self-experienced fatigue was high and motivation was low. The time on task effect was more pronounced in older participants for mental fatigue, interaction ToT by Age: *F*_(2, 48)_ = 3.33, *p* = 0.061, $\eta_{p}^{2}=0.12$, but not for motivation ratings, *F*_(4, 48)_ = 0.069, *p* > 0.2, indicating that older adults experienced a stronger increase in mental fatigue than younger adults. On the other hand, for both measures some evidence for an interaction of age by task was found, fatigue: *F*_(3, 72)_ = 2.44, *p* = 0.108, $\eta_{p}^{2}$ = 0.09, motivation: *F*_(3, 72)_ = 3.23, *p* = 0.045, $\eta_{p}^{2}=0.12$, indicating more impact of the Simon task in younger compared to older adults.

### Behavioral data (simon task)

**Figure 3:**
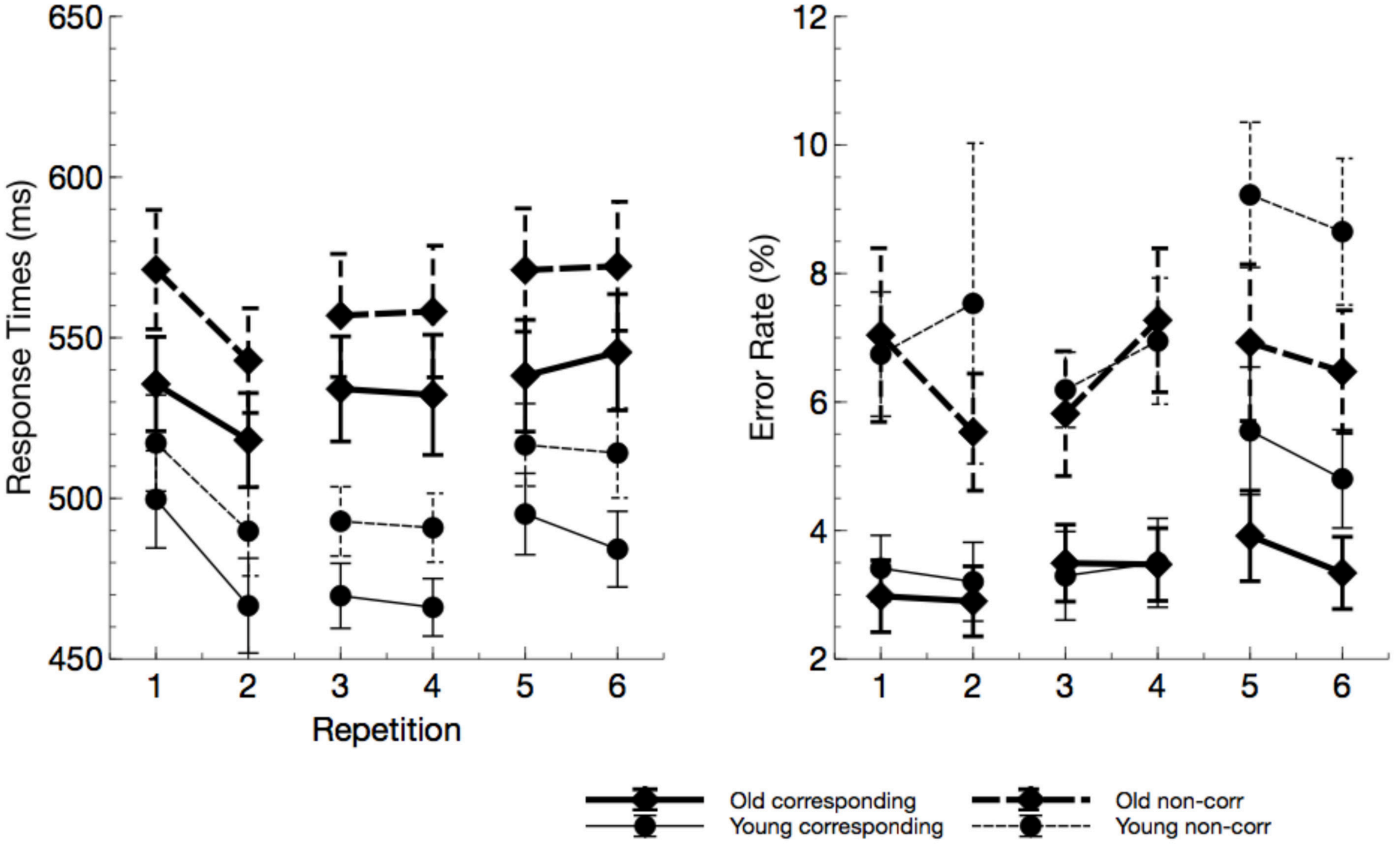
**Response times and error rates from the Simon task (“repetitions” reflect the factor sequence)**. Older adults responded slower but not less accurate. In particular in the last block, error rate in young participants markedly increased due to mental fatigue.

Older adults responded marginally slower than younger participants in the Simon task (see Figure 3), *F*_(1, 23)_ = 3.99, *p* = 0.058, $\eta_{p}^{2}=0.15$, and responses were faster for S-R corresponding trials, *F*_(1, 23)_ = 6.25, *p* < 0.001, $\eta_{p}^{2}=0.76$. No overall effect of ToT was found, *F*_(2, 46)_ = 2.16, *p* = 0.132, $\eta_{p}^{2}=0.09$, however, within blocks (Sequence), response times were faster after the physical task, *F*_(1, 23)_ = 7.67, *p* = 0.007, $\eta_{p}^{2}=0.25$. This phenomenon was most pronounced at the beginning of the experiment, interaction of ToT by Sequence: *F*_(2, 46)_ = 3.99, *p* = 0.007, $\eta_{p}^{2}=0.21$. No systematic variation of time on task parameters with age was found.

No age effect was found for error rates, *F*_(1, 23)_ = 0.40, *p* = 0.534, $\eta_{p}^{2}=0.02$, but there was an increase of error rates with non-corresponding trials, relative to corresponding trials, *F*_(1, 23)_ = 39.31, *p* < 0.001, $\eta_{p}^{2}=0.63$. Error rates slightly increased with ToT, *F*_(2, 46)_ = 5.16, *p* = 0.013, $\eta_{p}^{2}=0.18$. The latter effect was more pronounced in younger adults, interaction ToT by age: *F*_(2, 46)_ = 2.79, *p* = 0.079, $\eta_{p}^{2}=0.18$, indicating that younger participants committed more errors in the last block of the experiment.

### EEG data

#### Gravity frequency (GF), alpha, and theta power

**Figure 4:**
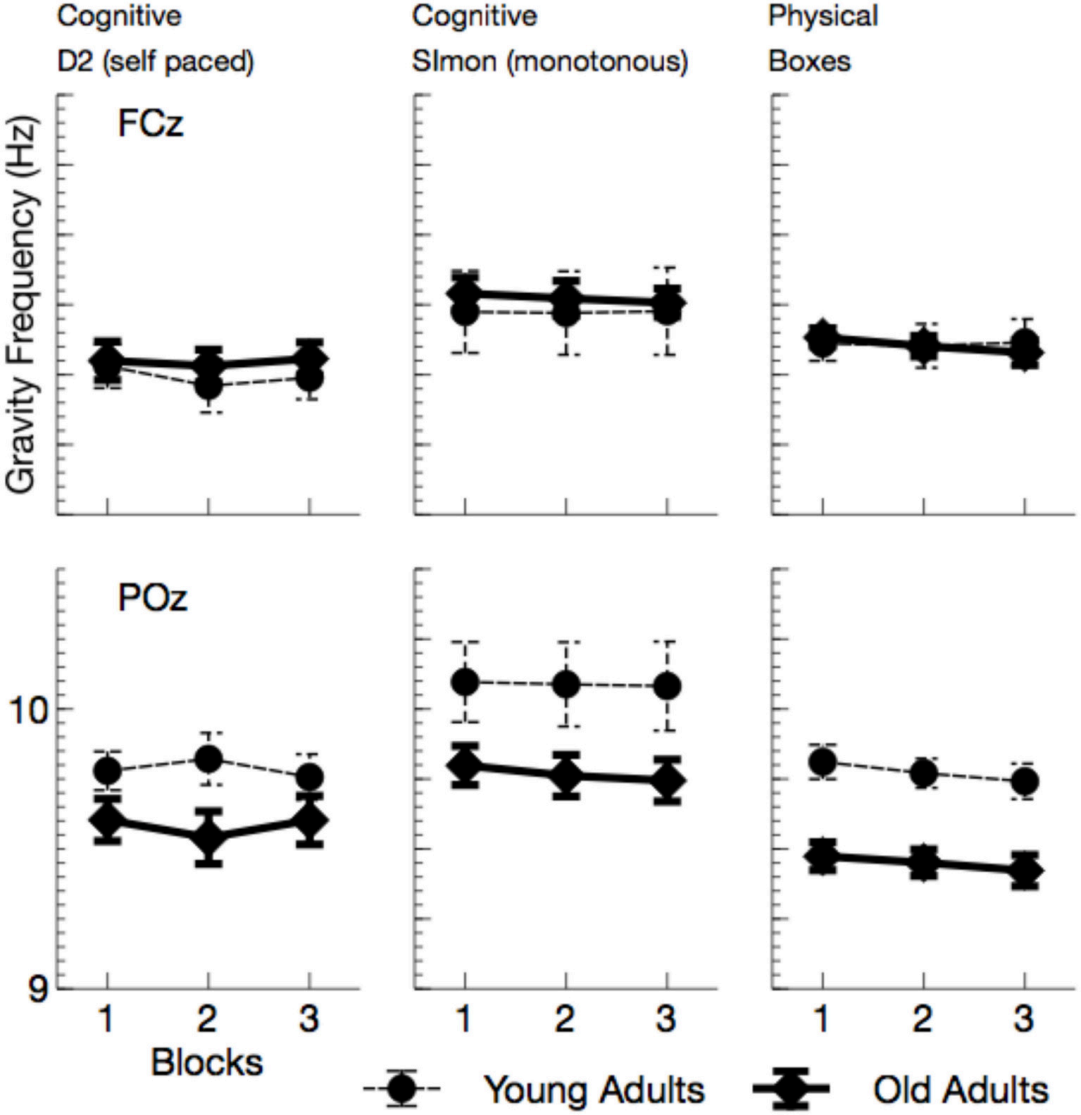
**Mean Gravity Frequency (with standard error of mean), separately for tasks and channels**. Most evident is the reduction of GF at posterior leads in all tasks.

GF (see Figure 4) did not overall vary with age, *F*_(1, 24)_ = 1.70, *p* = 0.205, $\eta_{p}^{2}=0.07$, but an interactions of age by channel *F*_(1, 24)_ = 26.91, *p* < 0.001, $\eta_{p}^{2}=0.53$, was found. No age effect was visible at the anterior lead, *F*_(1, 24)_ = 0.10, *p* > 0.05, whereas GF was reduced with higher age at the posterior electrode, *F*_(1, 24)_ = 8.12, *p* = 0.018, $\eta_{p}^{2}=0.25$. Also the effect of ToT did not reach significance, *F*_(1, 24)_ = 2.93, *p* = 0.200, $\eta_{p}^{2}=0.11$. However, GF strongly varied with the task performed, *F*_(2, 48)_ = 12.14, *p* < 0.001, $\eta_{p}^{2}=0.34$, and the effect of task was qualified by electrode position, *F*_(2, 48)_ = 13.74, *p* < 0.001, $\eta_{p}^{2}=0.36$. At frontal leads, GF was higher in the Simon task compared to the self-paced D2 task (D2), *F*_(1, 24)_ = 17.18, *p* < 0.001, $\eta_{p}^{2}=0.41$. Also, the physical task showed higher GFs than the D2 at the anterior lead, *F*_(1, 24)_ = 10.00, *p* = 0.008, $\eta_{p}^{2}=0.29$. At POz, again the Simon task evoked higher GFs compared to the D2, *F*_(1, 24)_ = 19.79, *p* < 0.001, $\eta_{p}^{2}=0.45$. At this electrode location no difference in GF was found between the two self-paced tasks (D2 and Boxes), *F*_(1, 24)_ = 2.31, *p* > 0.2.

**Figure 5:**
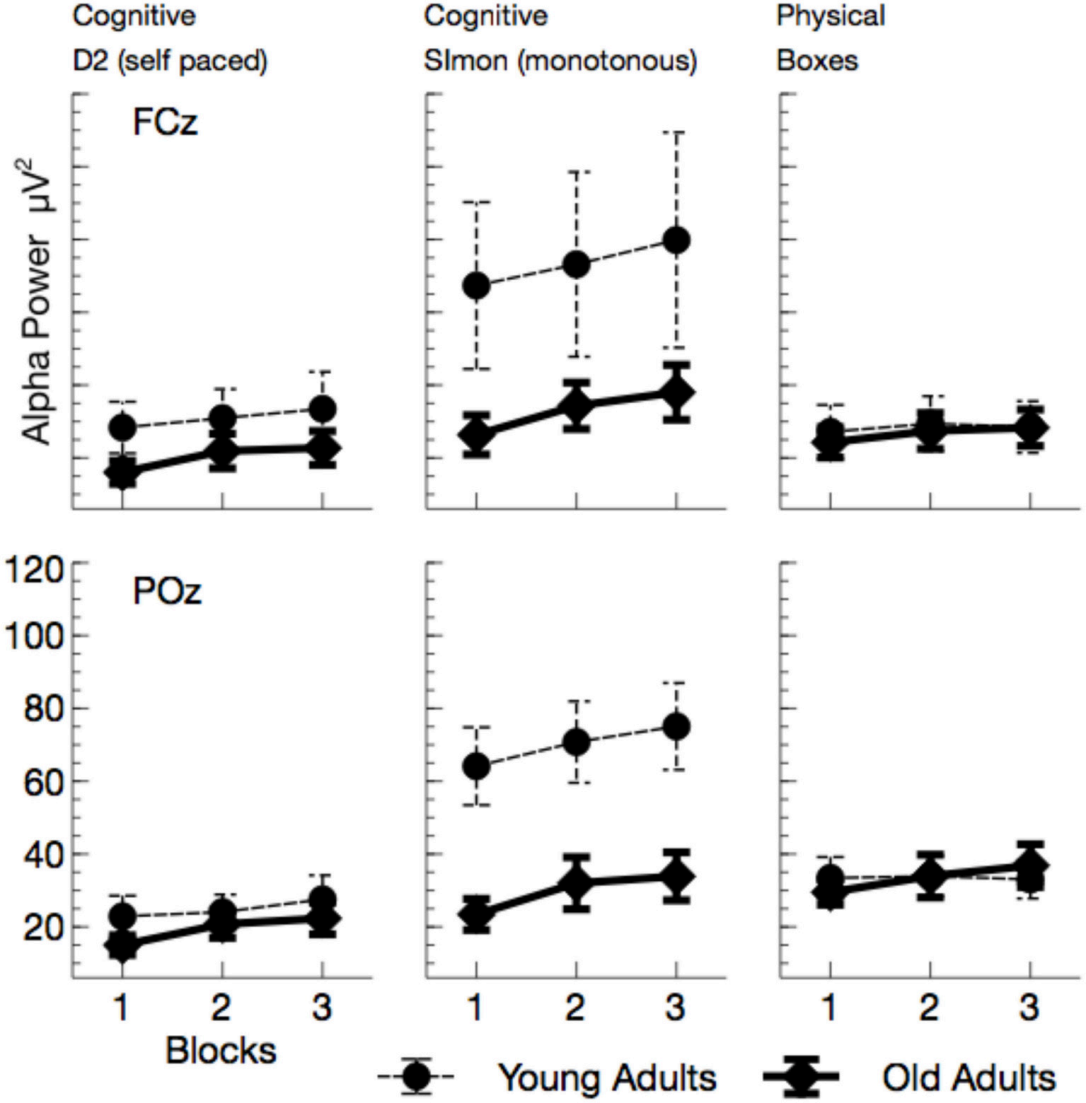
**Mean Alpha power (with standard error of mean), separated for tasks and channels**. Besides a general effect of ToT, younger adults show a massive increase in alpha power in the monotonous Simon task.

Alpha power (see Figures 5, **7**) increased with ToT, *F*_(1, 24)_ = 17.76, *p* < 0.001, $\eta_{p}^{2}=0.43$ and varied with task, *F*_(2, 48)_ = 10.96, *p* < 0.001, $\eta_{p}^{2}$ = 0.31. Pairwise comparisons revealed that in the cognitive tasks, alpha power was higher in the monotonous Simon task than in the self-paced D2 task, *F*_(1, 24)_ = 15.40, *p* = 0.002, $\eta_{p}^{2}=0.39$. Comparing the two self-paced tasks (D2 and Boxes), alpha power was higher when participants performed the physical task, *F*_(1, 24)_ = 18.18, *p* < 0.001, $\eta_{p}^{2}=0.43$. The effect of task was modulated both by age, *F*_(2, 48)_ = 5.81, *p* = 0.012, $\eta_{p}^{2}=0.19$, and by ToT, *F*_(2, 48)_ = 6.97, *p* = 0.004, $\eta_{p}^{2}=0.23$.

While ToT effects were obtained for both cognitive tasks [D2: *F*_(1, 24)_ = 17.69, *p* < 0.001, $\eta_{p}^{2}=0.42$; Simon: *F*_(1, 24)_ = 14.72, *p* = 0.003, $\eta_{p}^{2}=0.38$; Boxes: *F*_(1, 24)_ = 4.44, *p* = 0.138, $\eta_{p}^{2}=0.16$], the age effect was restricted to the Simon task. Alpha power was enhanced in younger adults, *F*_(1, 24)_ = 4.83, *p* = 0.076, $\eta_{p}^{2}=0.17$.

**Figure 6:**
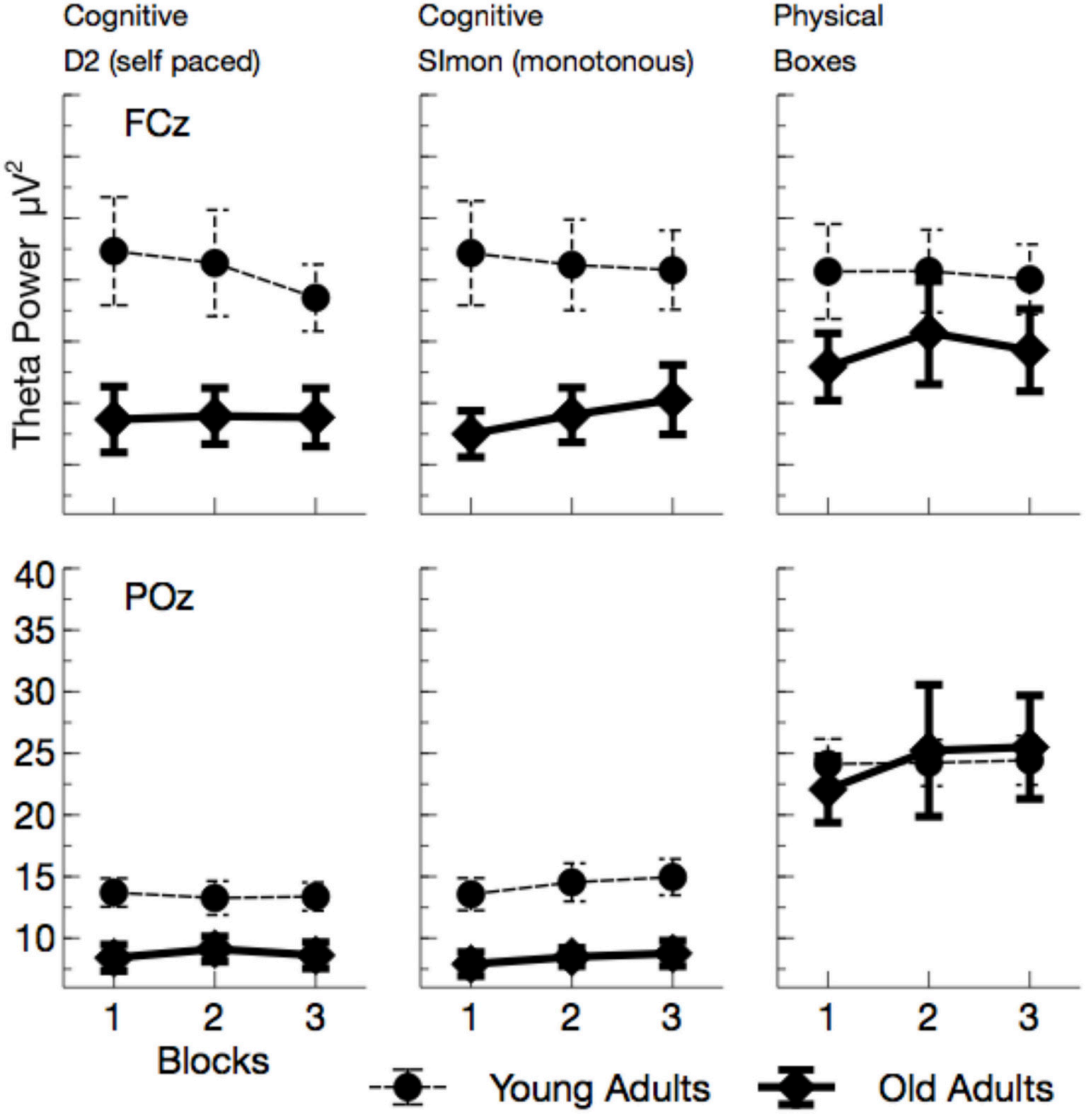
**Mean Theta power (with standard error of mean), separated for tasks and channels**. Most prominently, frontal Theta was reduced in older adults across all tasks. At POz, reduction of Theta power in older adults was restricted to cognitive tasks.

**Figure 7:**
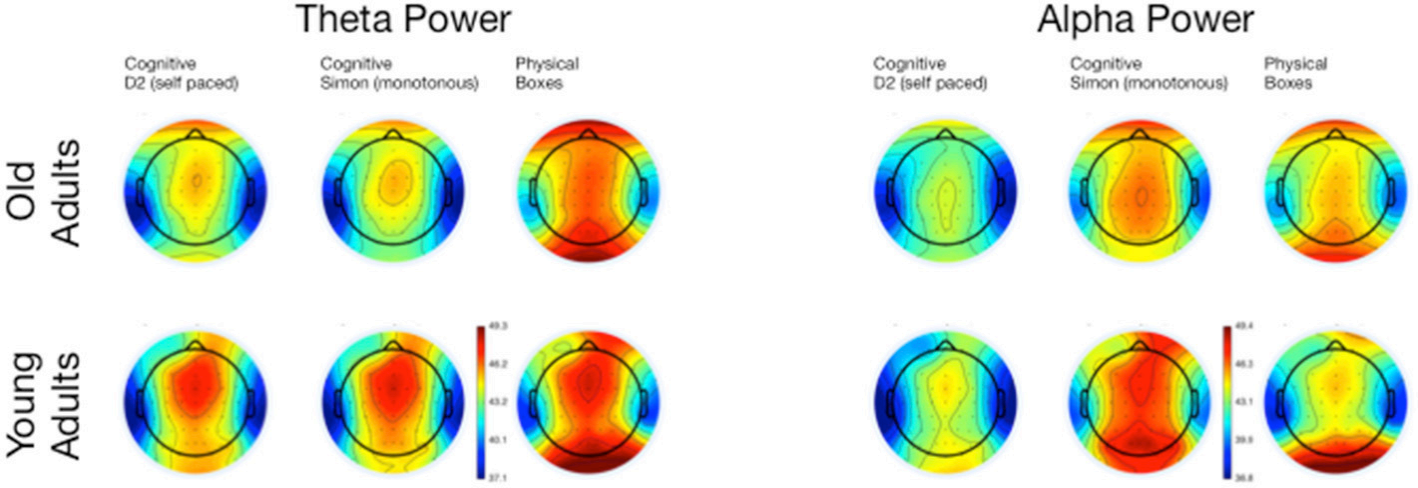
**Topographical maps of Theta and Alpha Power for the three tasks, separated for young and old adults**.

Theta power (see Figures 6, 7) was reduced in older adults, *F*_(1, 24)_ = 5.29, *p* = 0.030, $\eta_{p}^{2}=0.18$, and varied with the task performed, *F*_(2, 48)_ = 26.64, *p* < 0.001, $\eta_{p}^{2}=0.53$. Theta power did not differ between the two cognitive tasks (D2 and Simon), *F*_(1, 24)_ = 0.19, *p* > 0.5, but was markedly increased in the physical task compared to the self-paced cognitive D2 task, *F*_(1, 24)_ = 37.66, *p* < 0.002, $\eta_{p}^{2}=0.61$. No effect of ToT was observed, *F*_(1, 24)_ = 0.20, *p* > 0.5. Task effects were more pronounced at frontal leads, *F*_(2, 48)_ = 46.81, *p* < 0.001, $\eta_{p}^{2}=0.66$, and varied across age groups, *F*_(2, 48)_ = 3.67, *p* = 0.033, $\eta_{p}^{2}=0.13$. Significant age effects were only observed in the cognitive tasks, D2: *F*_(1, 24)_ = 8.96, *p* = 0.018, $\eta_{p}^{2}=0.27$, Simon: *F*_(1, 24)_ = 10.48, *p* = 0.012, $\eta_{p}^{2}=0.30$, but not in the physical task, *F*_(1, 24)_ = 0.56, *p* > 0.5.

#### Blink-related desynchronization/synchronization (ERD/ERS) of the EEG

**Figure 8:**
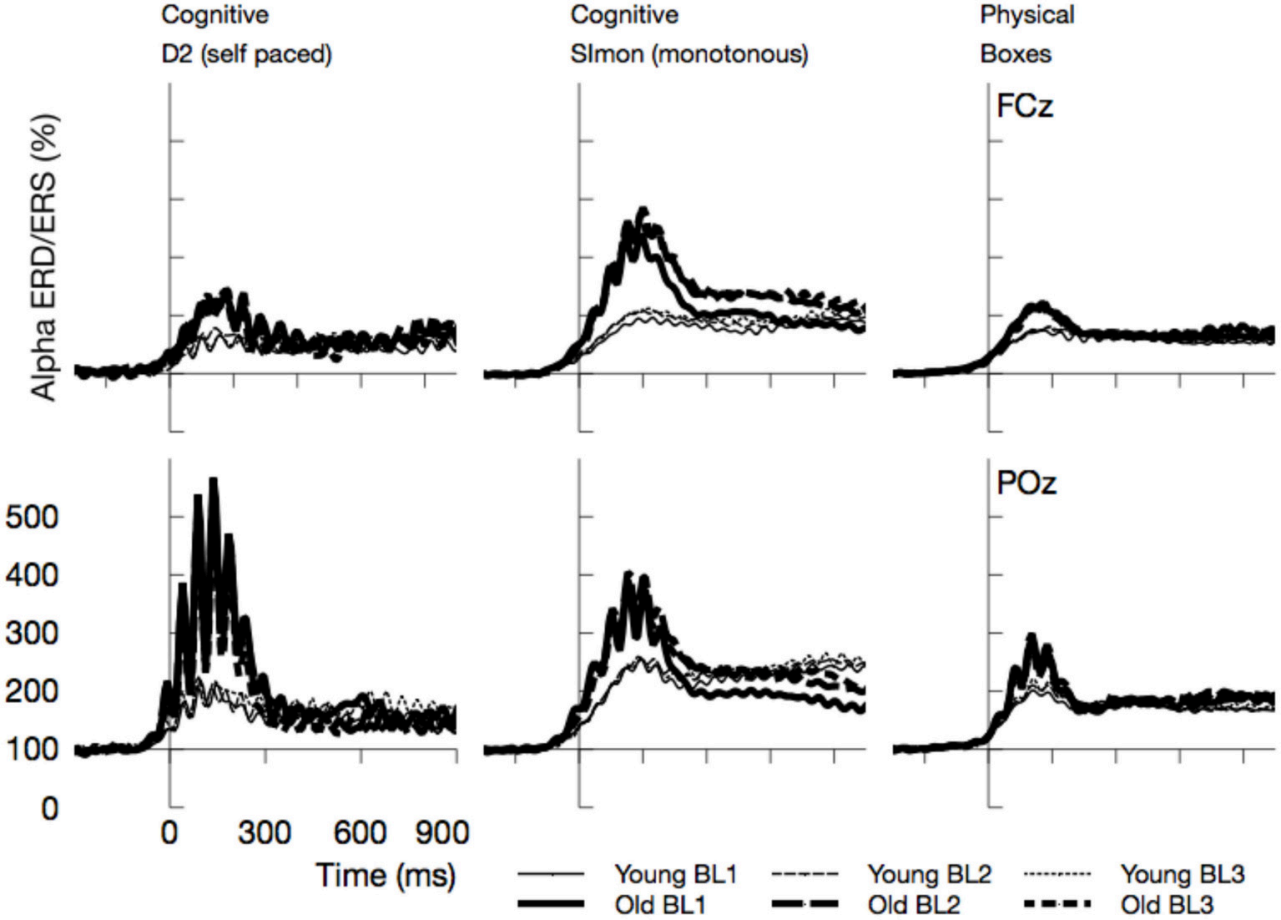
**Time course of Alpha synchronization for the three tasks and two channels, superposed for the three Blocks in the experiment (BL1, BL2, BL3)**. Data are time locked to the maximum of vEOG activation during the blink. Most prominently, Alpha synchronization was more pronounced in older adults.

As depicted in Figure 8, alpha activity synchronized after the eyes were opened. This effect was strongly modulated by experimental factors and differed across age groups. In the following, statistics will be reported for the mean ERD/ERS in the time window between 0 and 300 ms after the maximum of the blink in the EOG.

##### Alpha ERD/ERS

**Figure 9:**
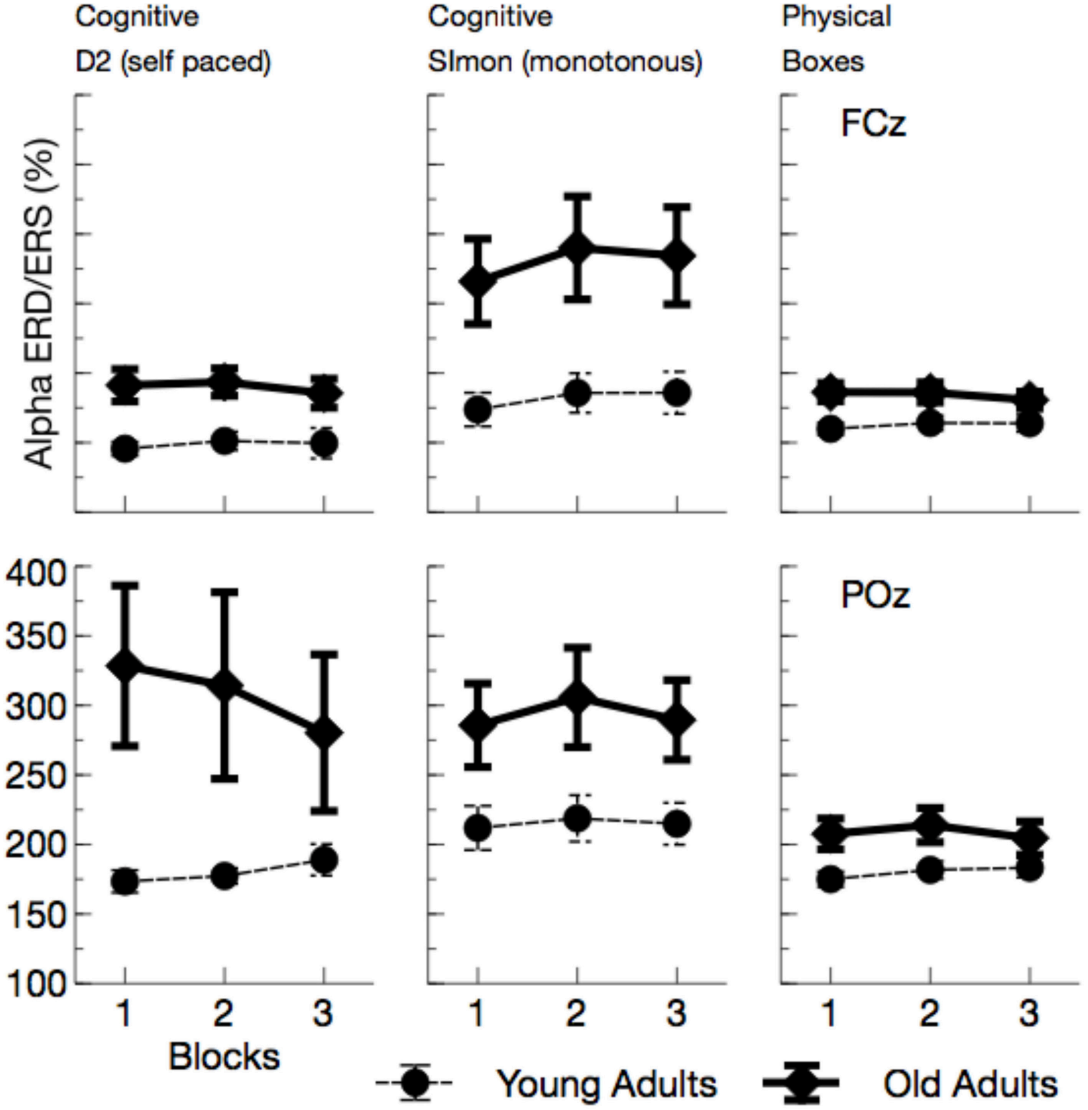
**Mean Alpha synchronization in a time window between 0 and 300 ms after the maximum of the eye-blink activity in the vEOG**. Enhanced Alpha synchronization in older adults was visible in all tasks. Due to effects of ToT, ERS/ERD measures became more similar between age groups with the duration of the ongoing experiment.

Event-related synchronizations in the alpha band (see Figures 8, 9) after the blink were enhanced for older adults, *F*_(1, 24)_ = 9.81, *p* = 0.005, $\eta_{p}^{2}=0.29$, and varied with the task performed, *F*_(2, 48)_ = 6.97, *p* = 0.002, $\eta_{p}^{2}=0.23$. Pairwise comparisons of tasks, however, did not show any significant effects [Simon vs. D2, *F*_(1, 24)_ = 3.95, *p* = 0.118, $\eta_{p}^{2}=0.14$, D2 vs. self-paced physical task, *F*_(1, 24)_ = 2.21, *p* = 0.300, $\eta_{p}^{2}=0.08$]. In the overall analysis also a number of interactions was observed, Age by ToT: *F*_(1, 24)_ = 5.91, *p* = 0.023, $\eta_{p}^{2}=0.20$, Age by Task by Channel: *F*_(2, 48)_ = 3.93, *p* = 0.026, $\eta_{p}^{2}=0.14$, Age by Channel by ToT: *F*_(1, 24)_ = 4.38, *p* = 0.047, $\eta_{p}^{2}=0.15$, Age by Task by channel by ToT: *F*_(2, 48)_ = 3.52, *p* = 0.038, $\eta_{p}^{2}=0.13$, indicating that ERS was modulated by all experimental factors. ERS systematically increased with ToT in younger adults, *F*_(1, 12)_ = 11.25, *p* = 0.012, $\eta_{p}^{2}=0.48$, but not in older ones, *F*_(1, 12)_ = 1.49, *p* = 0.492, $\eta_{p}^{2}=0.11$. Post-tests separate for each task revealed no significant interactions of Age by ToT [D2: *F*_(1, 24)_ = 4.72, *p* = 0.120, $\eta_{p}^{2}=0.16$, Boxes: *F*_(1, 24)_ = 5.47, *p* = 0.084, $\eta_{p}^{2}=0.19$, Simon task, *F*_(1, 24)_ = 0.09, *p* > 0.5].

##### Theta ERD/ERS

**Figure 10:**
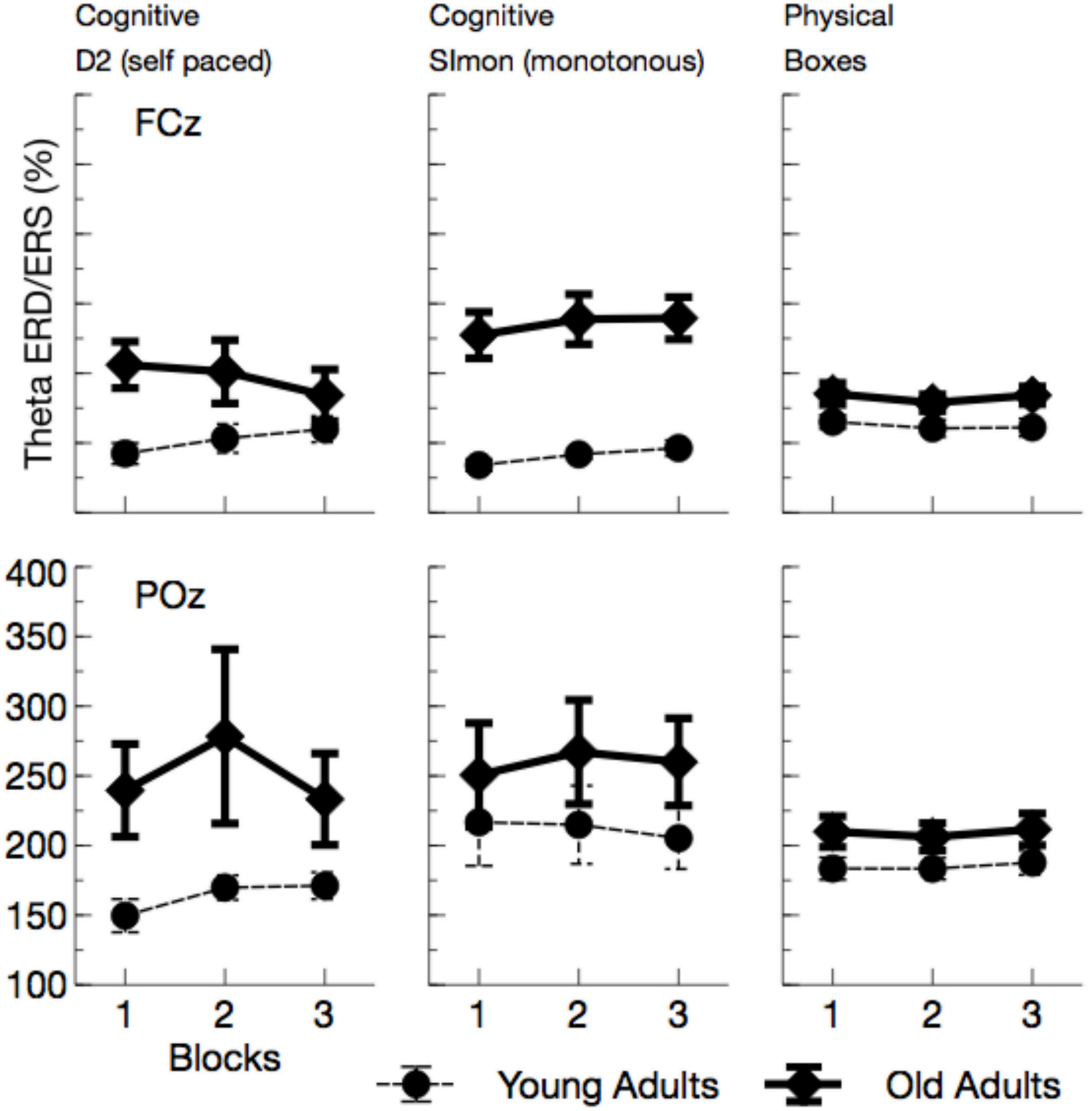
**Mean Theta synchronization in a time window between 0 and 300 ms after the maximum of the eye-blink activity in the EOG**. Also in this frequency band, more synchronization was observed in older adults. As for Alpha ERS/ERD a kind of convergence across age groups appeared, at least for the self-paced cognitive D2 task.

Overall, Theta ERS (see Figure 10) was enhanced in older adults, *F*_(1, 24)_ = 7.52, *p* = 0.011, $\eta_{p}^{2}=0.24$, and varied with the task performed, *F*_(2, 48)_ = 3.00, *p* = 0.059, $\eta_{p}^{2}=0.11$. Theta ERS was slightly higher in the Simon task compared to the self-paced D2 task, *F*_(1, 24)_ = 4.31, *p* = 0.049, $\eta_{p}^{2}=0.15$, but did not differ between the two self-paced tasks, *F*_(1, 24)_ = 0.21, *p* = 0.652, $\eta_{p}^{2}=0.01$. The effect of task was modulated by a number of other variables, Age by Channel by Task: *F*_(2, 48)_ = 3.01, *p* = 0.059, $\eta_{p}^{2}=0.11$, Age by Task by ToT: *F*_(2, 48)_ = 5.69, *p* = 0.006, $\eta_{p}^{2}=0.19$, Channel by Task by ToT: *F*_(2, 48)_ = 5.38, *p* = 0.008, $\eta_{p}^{2}=0.18$. All of those interactions reached significance in the young group, but failed to do so in older adults, reflecting the fact that Theta ERS strongly increased in variance in older adults. *Post-hoc* Tests revealed evidence toward an increase in Theta ERS for older adults in all tasks, D2: *F*_(1, 24)_ = 5.00, *p* = 0.105, $\eta_{p}^{2}=0.17$, Simon: *F*_(1, 24)_ = 7.21, *p* = 0.039, $\eta_{p}^{2}=0.23$, Boxes: *F*_(1, 24)_ = 5.10, *p* = 0.099, $\eta_{p}^{2}=0.18$. An interaction of Age by ToT was only observed in the self-paced cognitive task, D2: *F*_(1, 24)_ = 7.31, *p* = 0.036, $\eta_{p}^{2}=0.23$, Simon: *F*_(1, 24)_ = 0.94, *p* > 0.5, Boxes: *F*_(1, 24)_ = 0.00, *p* > 0.5.

In sum, the EEG data showed a pattern that is well comparable to previous experimental settings. Alpha power increased with ToT (see Wascher et al., 2014b) and showed a marked reduction in older adults. The latter effect, however, was restricted to the monotonous cognitive task that resembled most a regular cognitive experiment. Theta power was reduced in older adults and also systematically varied with the task performed. Age-related effects in this measure were more pronounced in cognitive tasks. Finally, a strongly enhanced synchronization of both frequency bands was observed when ERS/ERD were investigated time-locked to the blink maximum in the EEG.

## Discussion

In the present study, participants simulated a short (4–5 h) working shift in the post room of a wholesales house. They moved parcels and interacted with a computer in a repetitive sequence. The cognitive tasks on the computer were either repetitive (and rather monotonous) or self-paced. The design of the study was inspired by the real workflow in this particular working environment. During the entire shift, the EEG of the participants was recorded by mobile EEG equipment that did not restrict free movement and thus allowed natural behavior at any moment.

On average, subjectively experienced fatigue remained rather stable in younger adults during the entire shift, but increased for older adults. For both groups, however, fatigue was highly related to the task performed. After the monotonous computer task, fatigue ratings were substantially increased compared to the physical task. This finding nicely stresses the role of monotony for the experience of mental fatigue, which was more pronounced in younger adults who were obviously subjectively more affected by the monotonous task compared to older participants. These effects go along with a local decline in motivation in the monotonous cognitive task that was found only in the younger participants.

The enhanced impact of monotony upon younger participants is also nicely mirrored in behavioral and neurophysiological data. With increasing time on task, error rates but not response times increased in younger adults. At the end of the working shift, this led to an accuracy in the Simon task that was even lower in the younger, than in the older participants. Considering the EEG, younger participants showed markedly increased alpha activity in this particular task. Referring to the assumption that high alpha power is related to an idle state of the attentional system (Hanslmayr et al., 2012), younger adults might have switched to a state of attentional withdrawal (see Wascher et al., 2014b).

Within the theoretical framework described in the Section Introduction, in which mental fatigue may result either from cognitive overload or from mental underload (May and Baldwin, 2009), the fatiguing factor in this case is a passive one (pTR), namely monotony and the decline of motivation that goes along with that. Older adults appear to deal better with monotony. Factors that made them tired were more widespread across tasks. We can't rule out that in particular the physical task was more demanding for older adults. Factors like central fatigue, i.e., a decrease of cognitive capabilities due to muscular strain (e.g., Davis, 1995; Blomstrand, 2001) might have influenced their behavior in terms of an active task-related (aTR) factor. Both, age (Müller et al., 2009) and central fatigue (Hilty et al., 2011) have been reported to go along with larger cortical phase synchronization. In particular early synchronizations in the EEG might indicate that older adults were far more driven by external signals (see Klimesch et al., 2002) compared to young participants (Zacks and Hasher, 1997; Lustig et al., 2007; Wascher et al., 2011, 2012). This is in accordance with a number of laboratory studies that showed amplified early EEG responses both in evoked potentials and in time frequency based analyses (e.g., Müller et al., 2009). This stronger impact of stimulation is assumed to be due to reduced executive cognitive control with increasing age that may affect numerous cognitive functions (Gazzaley et al., 2005, 2008; Grady et al., 2006). As a neurophysiological correlate for this deficit, reduced frontal theta activity has been discussed (Cummins and Finnigan, 2007) which was also found in the present study. This latter effect, however, was restricted to the computer-based cognitive tasks and disappeared when boxes had to be sorted. Thus, we can demonstrate that the decrease in theta power is not a global fact with increasing age, but rather is a task-dependent decline. Thus, cognitive tasks appear to be more demanding for older adults, because of deficient signal handling when information enters the system. Too much irrelevant information might be processed (Wascher et al., 2012) which is resource consuming. Therefore, mental fatigue in older adults is at least in parts related to the exhaustion of cognitive functions.

Finally, regarding effects of time on task, alpha activity showed the well-known pattern of increasing power. In contrast to pure laboratory experiments (Wascher et al., 2014b), no saturation is visible in any task in the present study. This phenomenon might be due to the alternation of tasks that interrupted monotony. In particular, the huge increase of alpha power in younger participants in the Simon task indicate that monotony was an important factor that drove alpha power. More interestingly, both measures of event-related synchronization/desynchronization showed convergence between age groups with time on task. When younger participants were impaired in particular by the passive task-related factor of monotony, a decline in motivation should go along with that. Reduced motivation is correlated with reduced activity in the frontal dopaminergic motivation system (Berridge and Robinson, 1998). An impairment of executive control functions was the consequence and lead to more stimulus-driven behavior. This transient state resembles the aging brain that lacks frontal activity due to physical decline (Bäckman et al., 2000).

Taken together, these results show that applying neural measures to a real life work situation provided substantial information about mechanisms and causes of mental fatigue in younger and older adults. The core results were highly comparable to laboratory studies and therefore, validity and reliability of data appears to be sufficient. The diversity of tasks additionally provided important insight into the meaning and usefulness of particular neurophysiological measures for neuro-ergonomics. Most importantly, blink-related activity in the EEG (Berg and Davies, 1988) was systematically changing with the task performed and with other experimental factors. As has been shown before (Wascher et al., 2014a), cognitive demands and cognitive strategies are reflected in these measures. Thus, it can be assumed that the re-opening of the eyes, after a blink has been executed, denotes a moment when new information enters the system, very similar to the presentation of a visual stimulus. This fact allows to measure event-related EEG analyses without any external stimulation. In particular in working situations, nothing has to be changed to the natural environment. Nevertheless, aspects of information processing can be specifically addressed.

In summary, the present study demonstrates that mobile EEG provides substantial information about information processing at the workplace and its alteration due to fatigue or age related aspects. The data pattern in self assessments, behavioral data, and neurophysiological measures nicely indicates that younger participants suffered before most from monotony. Passive task-related fatigue led to deficits in information processing with time on task. Older adults, on the other hand, were challenged from the very beginning of the work shift by altered information processing. Due to declined executive control mechanisms, their information processing was much more stimulus-driven. Thus, the active process of overcoming this deficit appears to play a major role for mental fatigue in older worker in this particular working situation. Addressing this issue when designing working environment and work flow could substantially improve life quality of employees.

## Author contributions

EW wrote the MS and analyzed data. HH contributed to the design of the study and recorded data. SK was involved in the development of the theoretical framework. SA helped developing methods of analysis. SG contributed with respect to the theoretical embedding of age related cognitive decline. TM provided theoretical aspects with respect to mental fatigue.

## Funding

This research was supported by funding from the German Social Accident Insurance Institution for the trade and logistics sector (BGHW), the Federal Labour Office (Bundesagentur für Arbeit) and the “Metro AG.”

### Conflict of interest statement

The authors declare that the research was conducted in the absence of any commercial or financial relationships that could be construed as a potential conflict of interest.
